# Research on the High-Precision Position Datum Long Baseline Coordinate Transfer Strategy Using BDS-3 High- and Low-Frequency Signals

**DOI:** 10.3390/s26165307

**Published:** 2026-08-21

**Authors:** Mingduan Zhou, Wenxuan Zhang, Haodong Cai, Zichun Wang, Shuzhan Xia, Qiao Song, Shiqi Lin, Lu Qin

**Affiliations:** School of Geomatics and Urban Spatial Informatics, Beijing University of Civil Engineering and Architecture, Beijing 102616, China; 202506020235@stu.bucea.edu.cn (H.C.); 202406020118@stu.bucea.edu.cn (Z.W.); 202402010114@stu.bucea.edu.cn (S.X.); 2108160124002@stu.bucea.edu.cn (Q.S.); 2108570425026@stu.bucea.edu.cn (S.L.); 2108570424058@stu.bucea.edu.cn (L.Q.)

**Keywords:** BDS-3, position datum, coordinate transfer, high-frequency signal, low-frequency signal, long baseline processing, network adjustment

## Abstract

**Highlights:**

**What are the main findings?**

- The high-precision position datum long baseline coordinate transfer strategy using BDS-3 B1C and B2a signals is the best among the BDS-3 schemes.
- The BDS-3 B1C/B2a strategy achieves an average high-frequency carrier-phase precision of 6.3 mm, a mean NRMS of 0.23, millimeter-level baseline vector formal-error RMS, and a 19.7 mm point difference at DCMS.

**What are the implications of the main findings?**

- Under the experimental conditions of this study, BDS-3 B1C/B2a provides a promising dual-frequency option for regional and larger-scale high-precision position datum long-baseline coordinate transfer.
- The proposed strategy links carrier-phase observations, baseline vector quality, and network-adjusted point transfer into a complete evaluation chain for BDS-3 position datum transfer.

**Abstract:**

High-precision long-baseline coordinate transfer is essential for maintaining spatial reference frames, and the modernized multi-frequency signals of BDS-3 provide new opportunities for this task. However, existing long-baseline network solutions still rely mainly on legacy frequency combinations, and quantitative evidence for pure new-frequency BDS-3 combinations in large-scale coordinate transfer remains limited. This study evaluates the applicability of BDS-3 high- and low-frequency signal combinations for long-baseline position datum transfer and investigates frequency-combination selection. Seven continuous stations were used to form 21 long baselines. Five dual-frequency schemes were tested, including four BDS-3 combinations, namely B1I/B3I, B1I/B2a, B1C/B2a, and B1C/B3I, and one GPS reference combination, L1/L5. Double-differenced ionosphere-free baseline processing and three-dimensional constrained network adjustment were applied. Performance was assessed using carrier-phase precision, normalized root mean square (NRMS), baseline vector quality, and point-transfer differences. The results show that the BDS-3 B1C/B2a new-frequency combination achieved the best overall consistency among the BDS-3 schemes, with an average high-frequency carrier-phase precision of 6.3 mm, a mean NRMS of 0.23, millimeter-level baseline vector formal-error RMS, and a 19.7 mm point difference at the unknown station DCMS. Given that the evaluation is based on seven consecutive days of observations, the long-term applicability of the proposed strategy requires further validation.

## 1. Introduction

A high-precision position datum provides the spatial foundation for geodesy, crustal deformation monitoring, engineering control surveying, intelligent transportation, natural resource monitoring, and regional geospatial services [1,2,3]. With the development of multi-system, multi-frequency, and multi-constellation GNSS observations, long-baseline and ultra-long-baseline networks have become an important means of transferring coordinate datums over regional and larger spatial scales [4,5]. The completion of the BeiDou Navigation Satellite System-3 (BDS-3) has further expanded this capability by providing global service coverage and modernized open-service signals [6,7]. In addition to legacy signals, such as B1I and B3I, BDS-3 provides new signals, including B1C, B2a, and B2b, which improve the flexibility of frequency-combination design for high-precision positioning and coordinate transfer [8,9].

Previous signal-level studies have shown that BDS-3 new-frequency signals have clear potential for high-precision GNSS applications. Zhang et al. [10] assessed BDS-3 B1C and B2a signals in terms of carrier-to-noise density ratio, pseudorange precision, and carrier-phase precision, and reported better observation quality than that of several BDS-2 legacy signals. Data-quality assessments of BeiDou signals at global IGS stations [11] and of the new BDS-3 signals [12] further confirmed the good quality of the B1C and B2a observations. Yue et al. [13], Zhu et al. [14], and Wang et al. [15] evaluated the data quality and precise point positioning performance of BDS-3 B1C/B2a observations, showing that these signals can support precise positioning applications. Geng et al. [16] compared BDS-3 B1C/B2a with B1I/B3I from the perspectives of observation quality, precise orbit determination, and PPP performance, while Li et al. [17] and Liu et al. [18] reported additional evidence for the quality and positioning capability of BDS-3 new signals. These studies provide an important basis for using BDS-3 new-frequency signals, but most of them focus on signal quality, PPP, or positioning performance rather than long-baseline position datum coordinate transfer.

At the modeling level, frequency combinations, stochastic models, and long-baseline processing strategies have received increasing attention. Li et al. [19,20] studied tropospheric decorrelation and ambiguity resolution for long-baseline GNSS networks, showing that atmospheric residuals and model constraints play a central role in long-baseline processing. Guo et al. [21] described precise orbit determination strategies for multi-constellation GNSS satellites, providing product-level support for precise baseline processing. Wu et al. [22], Xu et al. [23], and Wang and Pan [24] compared BDS multi-frequency PPP models and full-frequency observations, showing that frequency-combination selection must consider noise amplification, inter-frequency correlation, and model flexibility. In addition, the GAMIT/GLOBK processing system, tropospheric mapping functions, IERS conventions, and BDS-3 satellite antenna phase-center models provide the necessary modeling basis for high-precision long-baseline solutions [25,26,27,28,29,30]. These studies are essential for understanding the theoretical behavior of frequency combinations, but many remain at the model or PPP level and do not fully evaluate BDS-3 dual-frequency combinations in a unified, double-differenced long-baseline network.

In engineering applications, recent studies have tested BDS-3 in control surveying and position datum transfer. Lai et al. [31] applied BDS-3 to control surveying in Africa, and Li et al. [32] assessed the accuracy of BDS-3 in precise engineering control surveying. Zhou et al. [33,34] tested BDS-3 and BDS-2 dual-frequency observations in high-precision engineering survey networks and compared the performance of BDS-3, BDS-2, and combined BDS-2/BDS-3 systems for medium- and long-baseline control surveying. Zhou et al. [35] further investigated BDS-3 long-baseline position datum coordinate transfer and found that BDS-3 B1C/B2a can achieve carrier-phase precision comparable to GPS L1 and favorable baseline vector formal precision. Related studies also examined BDS-3 in engineering control surveying, combined BDS-2/BDS-3 applications, and the establishment and maintenance of geodetic coordinate reference frames [36,37,38,39,40,41]. These studies are closest to the present work, but they mainly focus on a limited number of combinations or combined BDS-2/BDS-3 configurations. A unified comparison of BDS-3 B1I/B3I, B1I/B2a, B1C/B2a, and B1C/B3I under the same processing strategy remains insufficient.

At the signal level, previous studies have mainly examined BDS-3 observation quality and error characteristics. In addition to carrier-to-noise density ratio and carrier-phase precision, multipath effects and observation-noise behavior are important for determining whether a signal can support stable long-baseline processing. Lu et al. [42] analyzed BDS-3 multipath characteristics and mitigation methods, and Cai et al. [43] further compared the measurement noise and multipath of GPS, BeiDou, GLONASS, and Galileo observations. Dai et al. [44] examined carrier-phase multipath behavior using sidereal filtering. Zheng et al. [45], Dong et al. [46], and Zhan et al. [47] further developed multipath mitigation models based on multi-GNSS PPP, multipath hemispherical maps, and trend-surface modeling. These studies indicate that multipath modeling can improve positioning stability, but most methods require sufficient historical observations or stable observing environments. Therefore, their conclusions cannot be directly transferred to short-period long-baseline coordinate-transfer networks.

In precise positioning, many studies have developed PPP, PPP-AR, RTK, and PPP-RTK algorithms for multi-frequency GNSS data. Li et al. [48] proposed a BDS multi-frequency PPP ambiguity-resolution method using new B2a/B2b/B2a + b signals and legacy B1I/B3I signals. Liu et al. [49] developed a phase observable-specific signal-bias model for BDS-3 multi-frequency PPP-AR, and Jiao et al. [50] studied mixed OSB estimation for multi-frequency PPP with receiver time-varying biases. Shi et al. [51] and Zhao et al. [52] further showed that inter-frequency bias and differential code-bias handling are important for PPP convergence and performance in constrained environments. In the RTK and PPP-RTK fields, Lyu et al. [53] proposed regional reference network augmentation for BDS PPP-AR, while Ge et al. [54] and Geng et al. [55] improved ambiguity resolution using fractional-cycle bias estimation. Guo et al. [56] further assessed multi-frequency PPP ambiguity resolution with Galileo and BDS-3 signals, and Zhang et al. [57] proposed a novel un-differenced PPP-RTK concept. Teunissen established the LAMBDA method for integer ambiguity resolution, and subsequent studies showed that triple-frequency observations can improve extra-wide-lane ambiguity fixing and cycle-slip repair [58,59,60]. Triple-frequency signals also benefit medium- and long-baseline RTK positioning with BDS-3 B1C/B2a observations [61]. These algorithms provide theoretical and methodological support for multi-frequency GNSS positioning, but their reliance on precise products, regional augmentation, or ambiguity-bias products differs from the double-differenced long-baseline network adjustment used in this study.

Bias-related studies further show that pseudorange variations, hardware delays, and antenna corrections affect the performance of BDS-3 frequency combinations. Wanninger and Beer [62] diagnosed satellite-induced code pseudorange variations in BDS and proposed an empirical correction method. Xie et al. [63] analyzed BDS-3 measurement quality and precise orbit determination performance, while Shi et al. [51] modeled BDS-2/BDS-3 single-frequency PPP with B1I and B1C signals. Fan et al. [64] estimated BDS differential code biases using triple-frequency uncombined PPP, showing that bias handling can affect high-precision positioning and ambiguity resolution. These studies show that BDS-3 performance is not controlled only by signal strength or nominal frequency design. It is also affected by satellite-end and receiver-end biases, antenna corrections, and product consistency. These factors are especially important when new and legacy BDS-3 frequencies are compared in the same long-baseline network.

Beyond positioning, BDS-3 new-frequency signals have also been extended to precise orbit determination, time transfer, satellite-based augmentation, and practical processing systems. He et al. [65] improved BDS-3 precise orbit determination using the B1C/B2a dual-frequency combination. Xu et al. [66] developed a BDS-3 five-frequency carrier-phase time-transfer model, and Su and Jin [67] developed triple-frequency carrier-phase time-transfer models for BDS-3. Yang et al. [68] and Xu et al. [69] evaluated BDS-3 PPP-B2b services for real-time precise point positioning. These studies show that BDS-3 new-frequency signals can support not only static positioning but also precise orbit determination, time transfer, and satellite-based augmentation. However, these applications usually depend on specialized products, real-time corrections, or service-specific signal links. They therefore do not directly answer how different BDS-3 high- and low-frequency combinations behave in a ground-based long-baseline position datum transfer network. Software manuals, network-adjustment systems, and geodetic textbooks provide practical implementation support, but they do not replace empirical evaluation of frequency combinations under a unified experimental design [25,70,71].

In summary, previous studies have demonstrated the potential of BDS-3 new-frequency signals from the perspectives of signal quality, error modeling, frequency-combination design, PPP/RTK algorithms, engineering surveying, and satellite-based services. However, three limitations remain. First, many studies focus on signal-level quality, PPP, RTK, or short- to medium-baseline positioning, while the propagation of observation errors into long-baseline network solutions and point-transfer results remains less well examined. Second, existing long-baseline studies mainly focus on a limited number of BDS-3 combinations or combined BDS-2/BDS-3 configurations; systematic comparisons among BDS-3 B1I/B3I, B1I/B2a, B1C/B2a, and B1C/B3I under a unified processing strategy are still limited. Third, previous evaluations often rely on a single indicator, such as carrier-phase precision, positioning RMS, or baseline repeatability. Long-baseline position datum coordinate transfer requires a complete evidence chain that links carrier-phase precision, NRMS, baseline vector component differences, baseline vector repeatability, baseline vector formal errors, and three-dimensional constrained network-adjustment results.

To address these limitations, this study evaluates the applicability of BDS-3 high- and low-frequency combinations for high-precision position datum long-baseline coordinate transfer. Four BDS-3 dual-frequency combinations are tested, including B1I/B3I, B1I/B2a, B1C/B2a, and B1C/B3I. The GPS L1/L5 combination is used as a relative reference scheme, not as an absolute truth benchmark. An experimental network consisting of seven continuous observation stations and 21 long baselines is constructed, and all schemes are processed using the same data sources, processing strategy, and constrained network-adjustment framework.

The main contributions of this study are as follows. First, this study introduces BDS-3 new-frequency signals into a long-baseline position datum transfer framework and evaluates four BDS-3 dual-frequency combinations under consistent processing conditions. Second, it compares the error behavior of different frequency combinations using a multi-indicator evaluation system that includes carrier-phase precision, NRMS, baseline vector component differences, repeatability, formal-error differences, and formal-error RMS. Third, it connects baseline processing with three-dimensional constrained network adjustment, thereby establishing a complete verification chain from observations to baseline vectors and final point-transfer results. These contributions provide empirical evidence for selecting BDS-3 frequency combinations in regional and larger-scale high-precision position datum transfer.

The remainder of this paper is organized as follows. Section 2 introduces the dual-frequency ionosphere-free baseline-processing model and the datum-transfer network-adjustment method. Section 3 describes the experimental network, data sources, processing strategy, five frequency-combination schemes, and baseline-processing results, including carrier-phase precision, NRMS, and baseline vector quality. Section 4 presents the network-adjusted point-transfer results. Section 5 discusses the error-propagation characteristics of different frequency combinations, their relationship with previous studies, and the limitations of this work. Section 6 summarizes the conclusions and outlines future work.

## 2. Methods

The proposed method transfers position datum coordinates over long baselines by linking BDS-3 multi-frequency observations, long-baseline network solutions, and datum-transfer network adjustment. Ground-based GNSS stations simultaneously receive BDS-3 satellite signals and record carrier-phase and pseudorange observations. In the Earth-centered Earth-fixed (ECEF) frame, the carrier-phase observation model is expressed as follows [72]:

(1) $$L_{i,j}=\rho_{i,j}+c(dt_{i}-dt_{j})-I_{i,j}+T_{i,j}+\lambda (N_{i,j}+\phi_{i}-\phi_{j})+\varepsilon_{i,j}$$

where $L_{i,j}$ is the carrier-phase observable; $\rho_{i,j}$ represents the geometric distance from satellite j to receiver i; c is the speed of light; $dt_{i}$ and $dt_{j}$ denote the clock offsets of the receiver and satellite, respectively; $I_{i,j}$ and $T_{i,j}$ are the ionospheric and tropospheric delays, respectively; $N_{i,j}$ signifies the integer ambiguity; $\phi_{i}$ and $\phi_{j}$ represent the initial phase biases or hardware delays at the receiver and satellite ends, respectively; and $\varepsilon_{i,j}$ is the measurement noise.

Because long-baseline coordinate transfer is affected by ionospheric delay, residual atmospheric errors, and correlations among baselines, dual-frequency ionosphere-free combinations were formed using high- and low-frequency observations. This step reduces the effect of first-order ionospheric delay on baseline processing. Long-baseline joint processing was then performed in network mode. Tropospheric delay was modeled using the Saastamoinen model and global mapping functions (GMF/VMF), and synchronous baseline vectors and their variance–covariance matrices were obtained. Single-day solution repeatability and the normalized root mean square (NRMS) were used to evaluate baseline-solution quality and the stability of different frequency-combination schemes.

**Figure 1:**
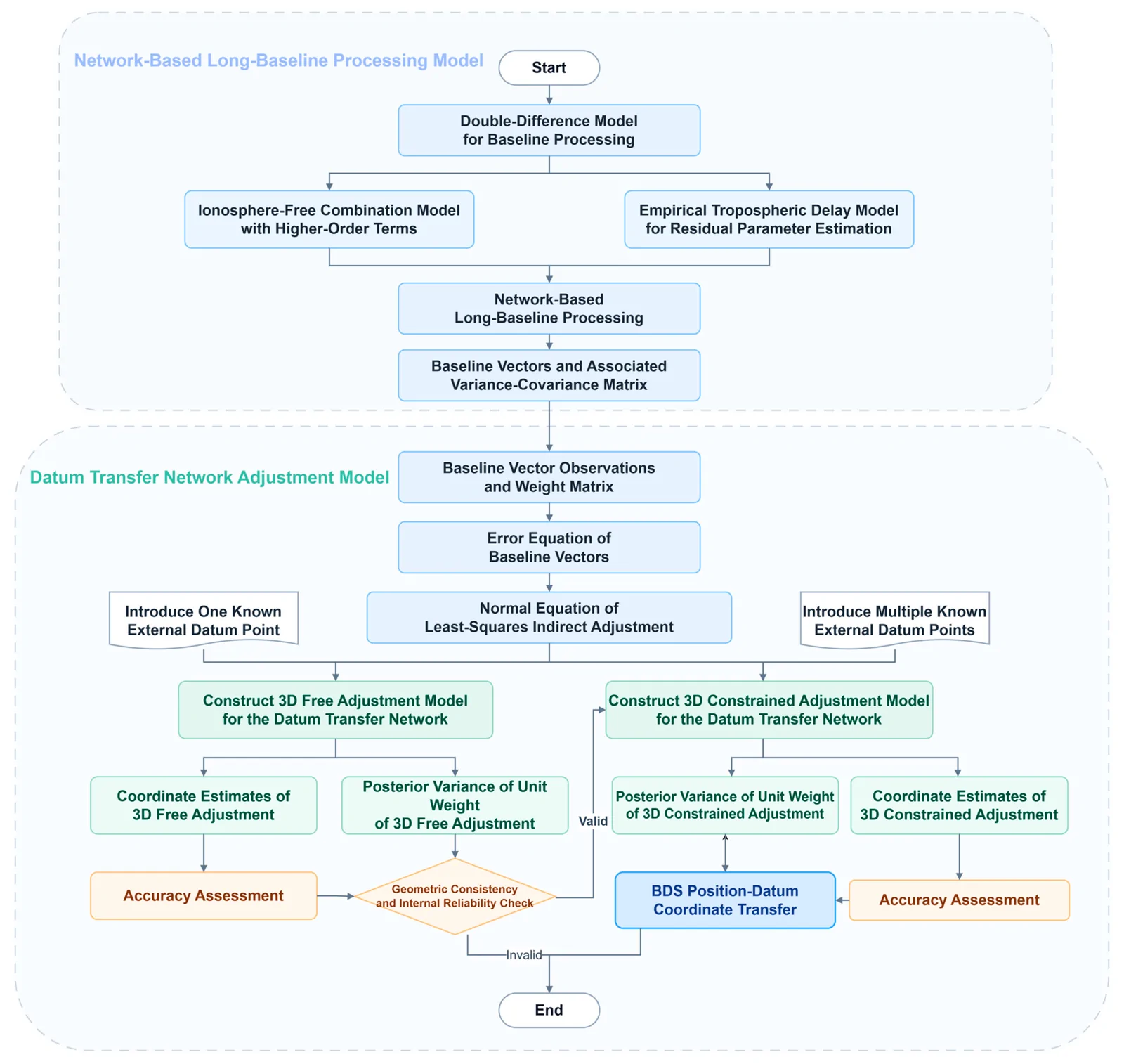
Workflow for BDS-3 high- and low-frequency signal position datum long-baseline coordinate transfer.

The baseline vectors and their variance–covariance matrices were then used as input observations for datum-transfer network adjustment. Known reference-station coordinates in the ITRF frame were introduced as external constraints, and a three-dimensional constrained network adjustment model was established using indirect adjustment. This model was used to estimate the coordinates and precision indicators of the unknown stations. The full workflow, therefore, links raw observations, baseline vectors, and final point-coordinate transfer results. The processing workflow for long-baseline position datum coordinate transfer using BDS-3 high- and low-frequency signals is shown in Figure 1.

### 2.1. Long-Baseline Resolution Model Based on Network Solution

The synchronized baseline vectors obtained from long-baseline resolution under the network solution mode can be expressed in matrix form as

(2) $$B={\left[\begin{array}{ccccc} b_{1} & \cdots & b_{k} & \cdots & b_{n} \end{array}\right]}^{T}$$

where $b_{k}={\left[\begin{array}{ccc} \Delta x_{k} & \Delta y_{k} & \Delta z_{k} \end{array}\right]}^{T}$ represents the k baseline vector observable, k=1,2,⋯,n. The variance–covariance matrix corresponding to these baseline vectors is given by

(3) $$\sum b=\left[\begin{array}{ccc} \sigma_{b_{1},b_{1}} & \cdots & \sigma_{b_{1},b_{n}}\\ \vdots & \ddots & \vdots \\ \sigma_{b_{n},b_{1}} & \cdots & \sigma_{b_{n},b_{n}} \end{array}\right]$$

As indicated by Equation (3), the variance–covariance matrices corresponding to individual baseline vectors are not necessarily zero matrices. This demonstrates that the baseline vectors resolved via the network solution mode can, to a certain extent, reflect the correlation among the baselines. The baseline vectors and their corresponding variance–covariance matrices formulated by Equations (2) and (3) can serve as prior inputs for the position datum transfer network adjustment model, functioning as baseline vector observables and their stochastic weighting matrices.

Let the baseline vector observable be denoted as $\Delta X_{ft}={\left[\begin{array}{ccc} \Delta X_{ft} & \Delta Y_{ft} & \Delta Z_{ft} \end{array}\right]}^{T}$, its residual vector as $V_{ft}={\left[\begin{array}{ccc} V_{\Delta X_{ft}} & V_{\Delta Y_{ft}} & V_{\Delta Z_{ft}} \end{array}\right]}^{T}$, the approximate coordinates of the stations to be determined as $X_{f}^{0}={\left[\begin{array}{ccc} X_{f}^{0} & Y_{f}^{0} & Z_{f}^{0} \end{array}\right]}^{T}$, the adjusted coordinate corrections of the stations as $\delta {\hat{X}}_{f}={\left[\begin{array}{ccc} \delta {\hat{X}}_{f} & \delta {\hat{Y}}_{f} & \delta {\hat{Z}}_{f} \end{array}\right]}^{T}$, the vector containing all post-adjustment station coordinate corrections as $\delta \hat{X}={\left[\begin{array}{ccccc} \delta {\hat{X}}_{1}^{T} & \cdots & \delta {\hat{X}}_{f}^{T} & \cdots & \delta {\hat{X}}_{m}^{T} \end{array}\right]}^{T}$ and the adjusted coordinates of the stations to be determined as ${\hat{X}}_{f}={\left[\begin{array}{ccc} {\hat{X}}_{f} & {\hat{Y}}_{f} & {\hat{Z}}_{f} \end{array}\right]}^{T}$. Here, f = 2, 3, …, m and t = 1, 2, …, m denote the tracking station indices, with f≠t and the hat symbol “^” signifies the estimated parameter values. For the f station to be determined, its coordinates are given by

(4) $${\hat{X}}_{f}=X_{f}^{0}+\delta {\hat{X}}_{f}$$

The error equation for the $\forall b_{k=ft}$ baseline vector is formulated as

(5) $$\left(\begin{array}{c} V_{\Delta X_{ft}}\\ V_{\Delta Y_{ft}}\\ V_{\Delta Z_{ft}} \end{array}\right)=-\left(\begin{array}{ccc} 1 & 0 & 0\\ 0 & 1 & 0\\ 0 & 0 & 1 \end{array}\right)\left(\begin{array}{c} \delta {\hat{X}}_{f}\\ \delta {\hat{Y}}_{f}\\ \delta {\hat{Z}}_{f} \end{array}\right)+\left(\begin{array}{ccc} 1 & 0 & 0\\ 0 & 1 & 0\\ 0 & 0 & 1 \end{array}\right)\left(\begin{array}{c} \delta {\hat{X}}_{t}\\ \delta {\hat{Y}}_{t}\\ \delta {\hat{Z}}_{t} \end{array}\right)-\left(\begin{array}{c} \Delta X_{ft}+X_{f}^{0}-X_{t}^{0}\\ \Delta Y_{ft}+Y_{f}^{0}-Y_{t}^{0}\\ \Delta Z_{ft}+Z_{f}^{0}-Z_{t}^{0} \end{array}\right)$$

Then, Equation (4) can be expressed in matrix form as

(6) $$V_{ft}=\left(\begin{array}{cc} -I & I \end{array}\right)\left(\begin{array}{c} {\hat{X}}_{f}\\ {\hat{X}}_{t} \end{array}\right)-L_{ft}$$

where I is a 3×3 identity matrix, and $L_{ft}$ represents the constant term matrix of the baseline vector observables.

### 2.2. Position Datum Transfer Network Adjustment Model

Given that n baseline vectors are obtained from the network-solution-based long-baseline resolution model, and the position datum transfer network adjustment model contains m stations, normal equations are constructed from the error equations of individual baseline vector observables. By accumulating the coefficients and constant terms of each individual error equation into their corresponding positions in the total error equation, the total error equation for all baseline vector observables can be expressed in matrix form as

(7) $$V=B\cdot \delta \hat{X}-L$$

where V, B, L, and represent the assembled matrix combinations of the corresponding terms from individual baseline vector error equations. According to the principle of the least-squares indirect adjustment method [71,73], the corresponding normal equation is derived as

(8) $$(B^{T}PB)\cdot \delta \hat{X}=B^{T}PL$$

where P is the weighting matrix of the baseline vector observables (i.e., the inverse of the corresponding variance–covariance matrix). Since Equation (8) exhibits a rank deficiency, rendering the system matrix non-invertible, an external position datum can be introduced to resolve this rank deficiency problem.

If a known external position datum is introduced by configuring the t-th station as the control data, the corresponding position datum equation is defined as

(9) $${\left(\begin{array}{ccc} \delta {\hat{X}}_{t} & \delta {\hat{Y}}_{t} & \delta {\hat{Z}}_{t} \end{array}\right)}^{T}={\left(\begin{array}{ccc} 0 & 0 & 0 \end{array}\right)}^{T}$$

Equation (9) can be expressed in matrix form as

(10) $$G_{\delta \hat{X}}^{T}\cdot \delta \hat{X}=0$$

where, $G_{\delta \hat{X}}^{T}=\left(\begin{array}{ccccc} 0 & \cdots & \underset{3\times 3}{I} & \cdots & 0 \end{array}\right)$. Simultaneously solving Equations (8) and establishes the 3D unconstrained network adjustment model for the position datum transfer network, which can be utilized to evaluate the geometric inconsistency among baseline vector observables and verify internal consistency precision. Applying the least-squares principle, the adjustment solution of the 3D unconstrained network adjustment model is derived as

(11) $$\delta \hat{X}={\left(B^{T}PB+G_{\delta \hat{X}}G_{\delta \hat{X}}^{T}\right)}^{-1}\cdot B^{T}PL$$

This yields the corresponding estimated coordinate parameters as

(12) $$\hat{X}=X^{0}+\delta \hat{X}$$

where $\hat{X}={\left(\begin{array}{ccccc} {\hat{X}}_{1} & \cdots & {\hat{X}}_{i\neq t} & \cdots & {\hat{X}}_{m} \end{array}\right)}^{T}$ represents the estimated coordinate parameter matrix of the stations to be determined. The corresponding precision estimation model is formulated as

(13) $$D_{\delta \hat{X}}={\hat{\sigma }}_{0}^{2}\times {\left(B^{T}PB+G_{\delta \hat{X}}G_{\delta \hat{X}}^{T}\right)}^{-1}\left(B^{T}PB\right){\left(B^{T}PB+G_{\delta \hat{X}}G_{\delta \hat{X}}^{T}\right)}^{-1}$$

where ${\hat{\sigma }}_{0}^{2}$ represents the estimated a posteriori variance of unit weight for the 3D unconstrained network adjustment model, given by

(14) $${\hat{\sigma }}_{0}^{2}=V^{T}PV/\left(3n-3m+3\right)$$

For BDS position datum coordinate transfer applications, it is typically necessary to introduce s known external position data as control alignment inputs, where 1<s<m and s∈Z. Assuming that the 3D spatial coordinates of the endpoints of a certain baseline vector are known in both the Earth-Centered Earth-Fixed (ECEF) coordinate system (e) and the local geodetic coordinate system (c), their coordinate transformation relationship is expressed as

(15) $${\left(\begin{array}{c} \Delta X_{ft}\\ \Delta Y_{ft}\\ \Delta Z_{ft} \end{array}\right)}_{e}=\left(1+\omega \right){\left(\begin{array}{c} \Delta X_{ft}\\ \Delta Y_{ft}\\ \Delta Z_{ft} \end{array}\right)}_{c}+R_{ft}\cdot \left(\begin{array}{c} \varepsilon_{X}\\ \varepsilon_{Y}\\ \varepsilon_{Z} \end{array}\right)$$

where, $R_{ft}=\left(\begin{array}{ccc} 0 & -\Delta Z_{ft} & \Delta Y_{ft}\\ \Delta Z_{ft} & 0 & -\Delta X_{ft}\\ -\Delta Y_{ft} & \Delta X_{ft} & 0 \end{array}\right)$. The transformed error equation for this baseline vector can be written as

(16) $$\begin{array}{l} \left(\begin{array}{c} V_{\Delta X_{ft}}\\ V_{\Delta Y_{ft}}\\ V_{\Delta Z_{ft}} \end{array}\right)=\left(\begin{array}{ccc} 1 & 0 & 0\\ 0 & 1 & 0\\ 0 & 0 & 1 \end{array}\right)\left(\begin{array}{c} \delta {\hat{X}}_{t}-\delta {\hat{X}}_{f}\\ \delta {\hat{Y}}_{t}-\delta {\hat{Y}}_{f}\\ \delta {\hat{Z}}_{t}-\delta {\hat{Z}}_{f} \end{array}\right)+\hat{\omega }\cdot {\left(\begin{array}{c} \Delta X_{ft}^{0}\\ \Delta Y_{ft}^{0}\\ \Delta Z_{ft}^{0} \end{array}\right)}_{c}+R_{ft}^{0}\cdot \left(\begin{array}{c} {\hat{\varepsilon }}_{X}\\ {\hat{\varepsilon }}_{Y}\\ {\hat{\varepsilon }}_{Z} \end{array}\right)+{\left(\begin{array}{c} \Delta X_{ft}^{0}\\ \Delta Y_{ft}^{0}\\ \Delta Z_{ft}^{0} \end{array}\right)}_{c}-{\left(\begin{array}{c} \Delta X_{ft}\\ \Delta Y_{ft}\\ \Delta Z_{ft} \end{array}\right)}_{e}\\ =\left[\begin{array}{cccc} -\underset{3\times 3}{I} & \underset{3\times 3}{I} & R_{ft}^{0} & \Delta X_{ft}^{0} \end{array}\right]\cdot \hat{\beta }-\left[{\left(\begin{array}{c} \Delta X_{ft}\\ \Delta Y_{ft}\\ \Delta Z_{ft} \end{array}\right)}_{e}-{\left(\begin{array}{c} \Delta X_{ft}^{0}\\ \Delta Y_{ft}^{0}\\ \Delta Z_{ft}^{0} \end{array}\right)}_{c}\right] \end{array}$$

where $\hat{\beta }={\left[\begin{array}{cccccccccc} \delta {\hat{X}}_{f} & \delta {\hat{Y}}_{f} & \delta {\hat{Z}}_{f} & \delta {\hat{X}}_{t} & \delta {\hat{Y}}_{t} & \delta {\hat{Z}}_{t} & {\hat{\varepsilon }}_{X} & {\hat{\varepsilon }}_{Y} & {\hat{\varepsilon }}_{Z} & \hat{\omega } \end{array}\right]}^{T}$ represents the design matrix of parameters to be estimated; $\Delta X_{ft}^{0}={\left[\begin{array}{ccc} \Delta X_{ft}^{0} & \Delta Y_{ft}^{0} & \Delta Z_{ft}^{0} \end{array}\right]}_{c}^{T}$ is the constant term vector derived from initial values of the baseline observables; and $R_{ft}^{0}$ is the matrix of substituted approximate values.

From Equation (16), the total error equation for all baseline vectors can be expressed in matrix form as

(17) $$V_{s}=B_{s}\cdot \hat{\beta }-L_{s}$$

where $B_{s}$ is a block matrix composed of m×n sub-matrices of size 3×3. From the s position data, the baseline configuration equations can be formulated in matrix form as

(18) $$G_{\delta {\hat{X}}_{s}}^{T}\cdot \hat{\beta }=0$$

where $G_{\delta {\hat{X}}_{s}}^{T}=\left[\begin{array}{ccccc} 0 & \cdots & I_{t_{i=s}} & \cdots & 0 \end{array}\right]$ is the constraint matrix, and $I_{t_{i=s}}$ is the 3×3 identity matrix corresponding to the $t_{i}$-th position datum within s with solving Equations (17) and (18) establishes the 3D constrained network adjustment model for position datum transfer, which can be utilized to analyze potential network scale and orientation variations caused by the introduction of external control data, as well as to implement BDS position datum coordinate transfer [17]. Applying the least-squares principle, the estimated coordinate parameters of the stations to be determined in the 3D constrained network adjustment model are obtained as

(19) $$\hat{\beta }={\hat{\beta }}_{0}+{\left(B_{s}^{T}PB_{s}+G_{\delta {\hat{X}}_{s}}G_{\delta {\hat{X}}_{s}}^{T}\right)}^{-1}\left(B_{s}^{T}PL_{s}\right)$$

The corresponding precision estimation model is expressed as

(20) $$D_{\hat{\beta }}={\hat{\sigma }}_{s}^{2}\cdot {\left(B_{s}^{T}PB_{s}+G_{\delta {\hat{X}}_{s}}G_{\delta {\hat{X}}_{s}}^{T}\right)}^{-1}\left(B_{s}^{T}PB_{s}\right){\left(B_{s}^{T}PB_{s}+G_{\delta {\hat{X}}_{s}}G_{\delta {\hat{X}}_{s}}^{T}\right)}^{-1}$$

where ${\hat{\beta }}_{0}$ and $\hat{\beta }$ represent the initial and estimated coordinate parameter matrices of the stations to be determined, respectively; $D_{\hat{\beta }}$ is the variance–covariance matrix corresponding to the estimated coordinate parameters; and ${\hat{\sigma }}_{s}^{2}$ signifies the estimated a posteriori variance of unit weight for the 3D constrained network adjustment model, given by

(21) $${\hat{\sigma }}_{s}^{2}=V_{s}^{T}PV_{s}/\left(3n-3m+3s\right)$$

## 3. Experiments

### 3.1. Experimental Scheme Design

**Figure 2:**
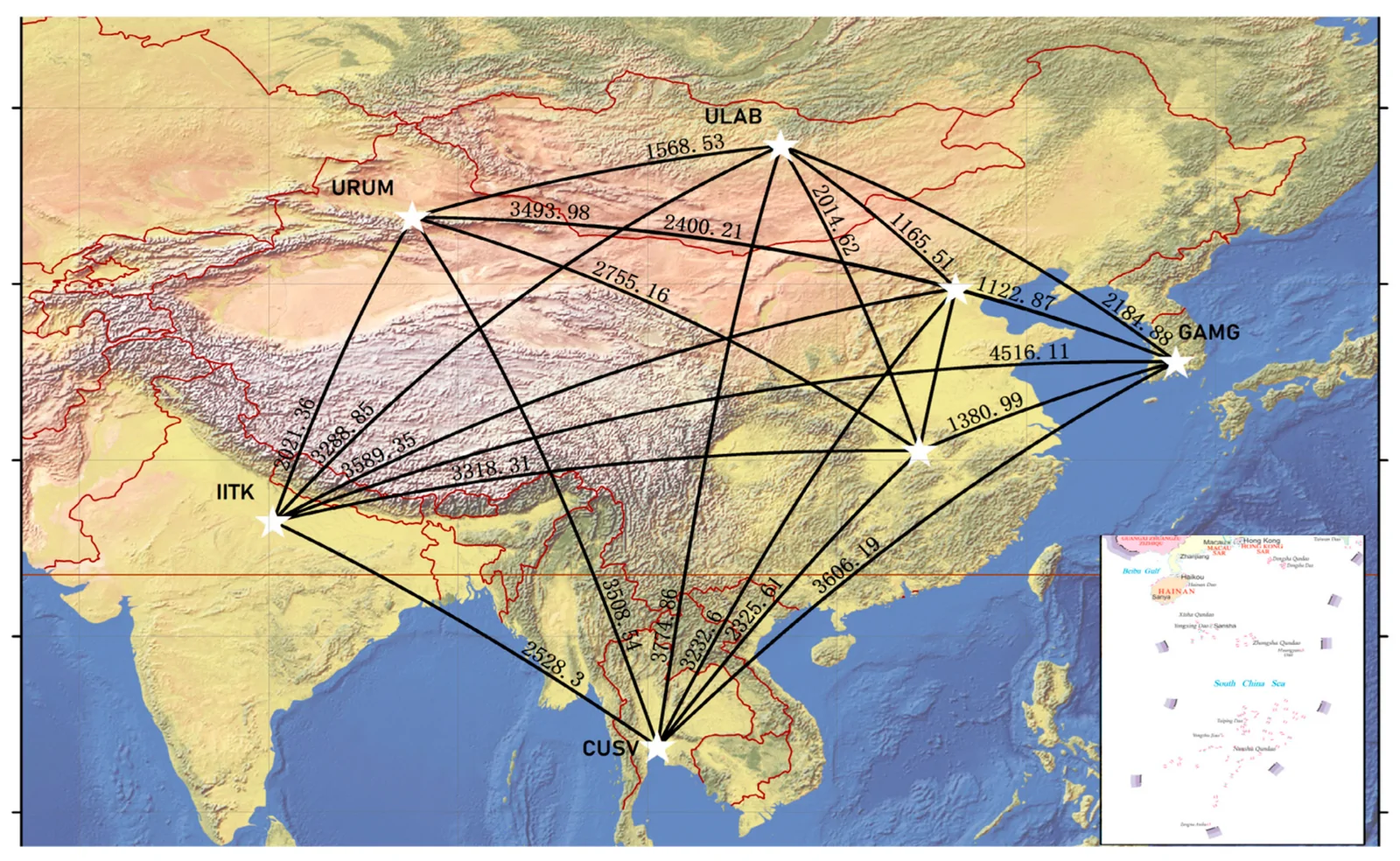
Experimental network for BDS position datum coordinate transfer. Baseline lengths are given in kilometers. The red line in the figure represents the Tropic of Cancer.

To evaluate the feasibility and effectiveness of the proposed high-precision long-baseline position datum coordinate-transfer strategy using BDS-3 high- and low-frequency signal combinations, an experimental network was established using six IGS tracking stations in China and surrounding regions and one self-deployed fixed station, DCMS. The six IGS stations are permanent tracking stations equipped with geodetic-grade multi-GNSS receivers, and DCMS is a fixed geodetic station newly deployed for this experiment. All selected IGS stations support the BDS-3 signals used in this study, including B1I, B3I, B1C, and B2a, as well as the GPS L1 and L5 signals used for the reference scheme. Although the BDS-3 B2b signal has demonstrated potential in precise point positioning and real-time services [68,69], B2b-based combinations (e.g., B1C/B2b) were not included in the present comparison. The adopted double-differenced processing strategy (GAMIT v10.71) provided uniform support for the four BDS-3 combinations evaluated in this study, whereas B2b-based processing was not yet available in this processing chain. The evaluation of B2b-involved combinations is therefore left to future work. As shown in Figure 2, the network forms 21 long-baseline vectors, with baseline lengths ranging from 1035.84 to 4516.11 km and an average length of 2611.08 km.

Seven 24 h observation sessions from day of year (DOY) 064 to 070 in 2026 were used. To ensure a consistent comparison among the five frequency-combination schemes, all schemes used the same stations, observation periods, data sources, and processing strategy. Observation files, broadcast ephemeris, and precise ephemeris were obtained from the GNSS Data Center of Wuhan University. The broadcast ephemeris were BRDC files for the corresponding DOY, and the precise ephemeris were SP3 orbit files. Before processing, the files were renamed according to GAMIT conventions, with long RINEX filenames converted to short filenames. The coordinate reference frame was ITRF2020, with the reference epoch set to 5 March 2026. Approximate station coordinates were provided by the lfile, and file, receiver type, antenna type, and antenna height were provided by station.info file.

Baseline processing was performed using GAMIT v10.71. The processing mode was set to BASELINE, the analysis type to 1-ITER, and the observation combination strategy to LC_AUTCLN. Thus, all long-baseline solutions were based on dual-frequency ionosphere-free combinations, while cycle-slip detection, observation editing, and ambiguity handling were performed using AUTCLN. In the LC_AUTCLN strategy, the wide-lane (WL) and narrow-lane (NL) ambiguities were resolved sequentially by AUTCLN, and all baseline solutions and evaluation metrics reported in this study are based on the final biases-fixed solutions. For each daily solution, the number of phase ambiguities in solution, WL ambiguities resolved by AUTCLN, and NL ambiguities fixed were extracted from the q-file, and the ambiguity-fixing rates of the five schemes were statistically analyzed. Each scheme used a 30 s sampling interval and 24 h observations, corresponding to 2880 epochs per day. The satellite elevation cutoff angle was set to 10°. Observation weighting followed an elevation-dependent model, and antenna phase-center corrections were applied using an azimuth-dependent and elevation-dependent model. During baseline processing, WUH2 was used as the tightly constrained station, with a 0.050 m constraint applied to each of the three coordinate components. The other stations were assigned loose constraints of 100 m in the three coordinate components to keep the constraint conditions consistent across all schemes.

Tropospheric delay was estimated using a piecewise linear model, and the vmf1grd.2026 grid file was used for tropospheric modeling. Zenith delay parameters were estimated every 2 h, with 13 parameters per day. The a priori zenith delay constraint was 0.50 m, the zenith delay variation constraint was 0.02 m/($\sqrt{h}$). Horizontal atmospheric gradients were estimated simultaneously, with two north–south and two east–west gradient parameters per day. The a priori gradient constraint was 0.03 m, and the gradient variation constraint was 0.01 m/($\sqrt{h}$). A priori meteorological information was read in the following order of priority: RINEX observation files, U-files, and the GPT model. First-order ionospheric delay was reduced using dual-frequency ionosphere-free combinations, with the ionospheric constraint set to 0.0 mm + 8.00 ppm.

The orbit and baseline-processing workflow followed the standard GAMIT procedure. The daily processing directory and table-file links were generated using links.day, and experiment files were generated using sh_makexp. The SP3 precise orbits were fitted using sh_sp3fit, satellite clock-related files were generated using makej, and makex, fixdrv, and the batch files were then run sequentially to generate X-files, driver files, and baseline solutions. After processing, sh_metutil was used to output station meteorological and zenith delay results, and sh_oneway was used to extract carrier-phase residual statistics. All schemes used the same preprocessing, orbit fitting, observation editing, tropospheric estimation, and quality-control settings; only the high- and low-frequency signals used to form the ionosphere-free combinations were changed.

Network adjustment was performed using CosaGPS v6.0. The synchronous baseline vectors and their precision information from GAMIT were used as input observations. CUSV, GAMG, IITK, ULAB, and URUM were used as external reference stations to establish a three-dimensional constrained network adjustment model. WUH2 was used as the check point, and DCMS was treated as the unknown station. To ensure a consistent comparison, the external reference stations, starting coordinates, adjustment model, and output indicators were kept identical for all five schemes. The performance of each frequency combination was evaluated using carrier-phase precision, NRMS, baseline vector quality, and network-adjusted point-transfer results.

The five experimental schemes were designed as follows:

Scheme A: BDS-3 B1I/B3I dual-frequency combination.
Scheme B: BDS-3 B1I/B2a dual-frequency combination.
Scheme C: BDS-3 B1C/B2a dual-frequency combination.
Scheme D: BDS-3 B1C/B3I dual-frequency combination.
Scheme E: GPS L1/L5 dual-frequency combination (relative reference scheme).

All schemes were processed using the same baseline-processing and network-adjustment workflow, allowing the effect of the frequency combination itself to be evaluated under consistent experimental conditions.

### 3.2. Baseline Processing Results

Four indicators were used to evaluate the long-baseline coordinate-transfer performance of the five frequency-combination schemes: high-frequency carrier-phase precision, normalized root mean square (NRMS), baseline vector quality, and point-transfer results. Baseline vector quality was further assessed using component differences, repeatability, formal-error differences, and formal-error RMS. Together, these indicators describe the performance of each scheme from observation quality to baseline stability and final coordinate transfer.

#### 3.2.1. Carrier-Phase Precision Analysis

**Figure 3:**
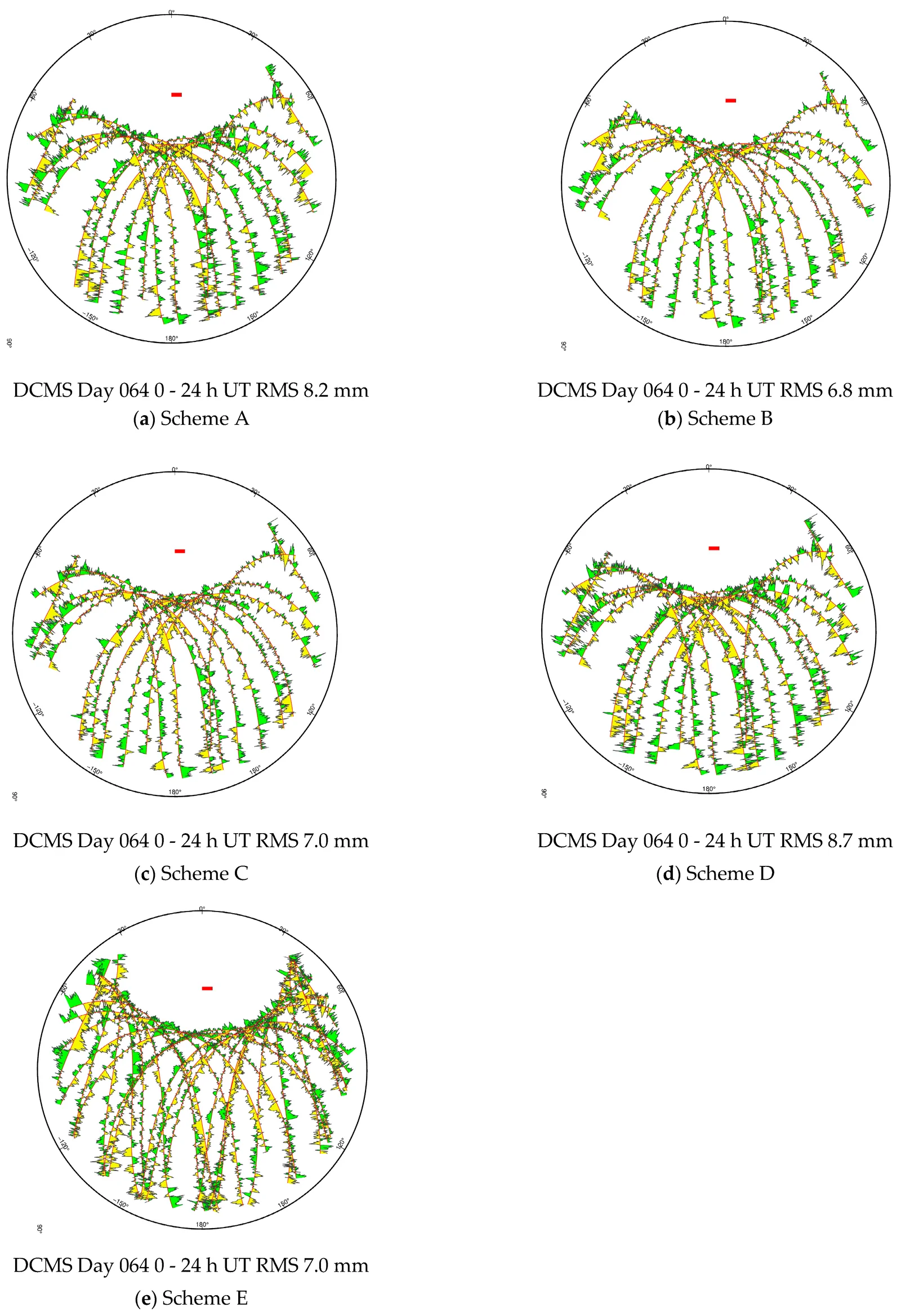
Carrier-phase sky plots at station DCMS on DOY 064 for Schemes A–E. Red curves denote satellite sky tracks, green and yellow shaded wiggles represent positive and negative carrier-phase residuals, respectively, and the central red rectangle indicates the residual scale reference.

The precision of the high-frequency carrier-phase residuals was analyzed for the seven stations in the experimental network from DOY 064 to DOY 070 in 2026. The high-frequency signals included BDS-3 B1I or B1C and GPS L1. Figure 3 shows the carrier-phase sky plots for all five schemes at station DCMS on DOY 064.

To evaluate precision over the full observation period, the carrier-phase residuals from DOY 064 to DOY 070 were statistically analyzed, and the results are summarized in Table 1. The statistical model used for carrier-phase precision analysis is given as follows [42]:

(22) $$\delta =\frac{1}{2}\sqrt{\sum_{i=1}^{p}\sum_{j=1}^{q_{i}-1}\nabla \Delta x_{ij}^{2}/\sum_{i=1}^{p}\left(q_{i}-1\right)}$$

where δ represents the precision of the carrier-phase observables; $\nabla \Delta x_{ij}^{2}$ denotes the double-differenced carrier-phase residual; i is the epoch index of the observation data; p signifies the total number of epochs; j represents the satellite PRN number; and $q_{i}$ is the number of visible satellites at epoch i.

As shown in Table 1, the mean carrier-phase precisions of the five schemes were 7.5, 6.4, 6.3, 7.5, and 6.1 mm for Schemes A–E, respectively. All schemes achieved millimeter-level carrier-phase precision. The GPS L1/L5 reference scheme, Scheme E, achieved the highest precision, with a mean value of 6.1 mm. Among the BDS-3 schemes, Scheme C, based on B1C, achieved a mean precision of 6.3 mm, which was comparable to Scheme B, based on B1I, at 6.4 mm. Both Schemes B and C performed better than Schemes A and D, which had mean precisions of 7.5 mm.

#### 3.2.2. NRMS Analysis

**Figure 4:**
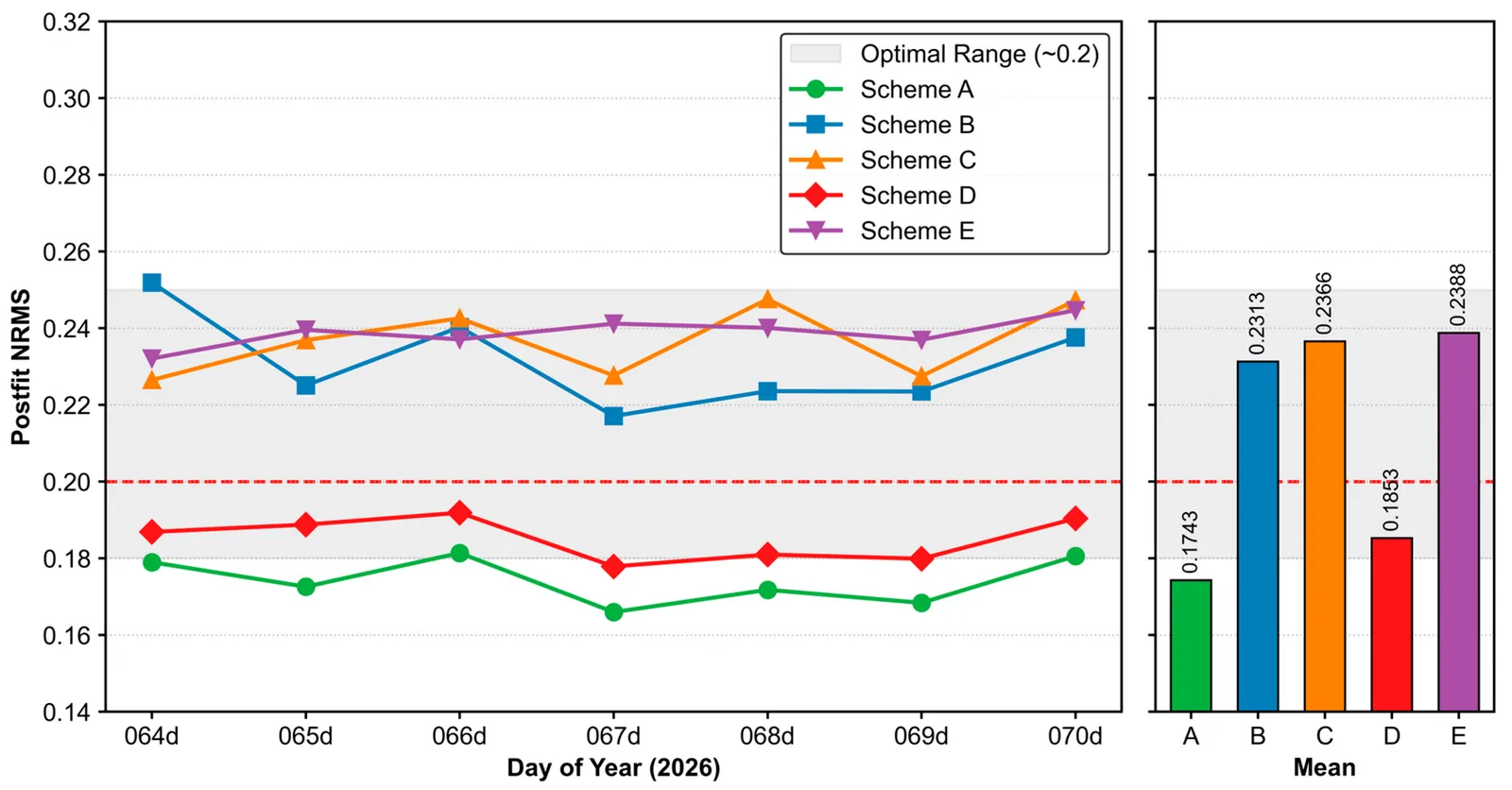
NRMS statistics for the five frequency-combination schemes. The red dashed lines represent the optimal range threshold (value = 0.2) for Postfit NRMS.

The normalized root mean square (NRMS) obtained from the LC_AUTCLN processing mode was used to assess baseline-solution quality. In general, an NRMS value below 0.5 indicates an acceptable baseline solution, while a value close to 0.2 indicates good processing quality [16]. Figure 4 shows the NRMS statistics for the five schemes from DOY 064 to DOY 070 in 2026.

As shown in Figure 4, the NRMS values for the five schemes and all observation days ranged from 0.17 to 0.25. The mean NRMS values of Schemes A-E were 0.17, 0.23, 0.23, 0.19, and 0.23, respectively. These values were close to 0.2, indicating that the baseline solutions for the five schemes were generally reliable. Scheme C and the GPS L1/L5 reference scheme, Scheme E, both had a mean NRMS of 0.23, suggesting that the baseline-solution quality of the BDS-3 B1C/B2a combination was comparable to that of the GPS L1/L5 reference scheme.

To confirm that these NRMS values characterize the biases-fixed solutions, the ambiguity-fixing statistics of the five schemes were extracted from the GAMIT q-files and are summarized in Table 2. As shown in Table 2, all five schemes achieved NL ambiguity-fixing rates above 90% (90.4%, 95.5%, 96.0%, 91.5%, and 94.1% for Schemes A–E, respectively). The high NL fixing rates indicate that the final baseline solutions of all schemes were reliable fixed solutions, and the NRMS values reported above therefore characterize the quality of the biases-fixed solutions. The WL fixing rates of the B2a-involved combinations (73.2% and 74.8% for Schemes B and C, respectively) were lower than those of the B3I-involved combinations (89.9% and 90.6% for Schemes A and D, respectively), and the underlying mechanisms are further discussed in Section 5.

#### 3.2.3. Baseline Vector Quality Analysis

Baseline vector quality was evaluated using four indicators: component differences, repeatability, formal-error RMS, and formal-error differences. This subsection first analyzes the component differences of the baseline vectors. The remaining indicators are discussed in the following subsections.

(1) Baseline Vector Component Differences

For each scheme, baseline vectors with the same station pair obtained from different observation days were averaged. Scheme E, based on GPS L1/L5, was used as the relative reference scheme. The differences in the north (N), east (E), up (U), and length (L) components were then calculated for each baseline vector. Table 3 presents the component differences for the six long baselines connecting DCMS with CUSV, GAMG, IITK, ULAB, URUM, and WUH2.

For Scheme A, the N-component differences ranged from −13.5 to 11.4 mm, with a mean of 2.6 mm. The E-component differences ranged from −21.3 to 12.8 mm, with a mean of −2.3 mm. The U-component differences ranged from −35.7 to 8.4 mm, with a mean of −13.9 mm, and the L-component differences ranged from −3.0 to 27.6 mm, with a mean of 14.2 mm. For Scheme B, the corresponding ranges were −9.7 to 23.1 mm for N, −15.7 to 12.3 mm for E, −47.6 to 6.7 mm for U, and −4.0 to 20.7 mm for L, with mean values of 9.3, −1.8, −16.4, and 9.3 mm, respectively.

For Scheme C, which corresponds to the BDS-3 B1C/B2a combination, the N-component differences ranged from −10.3 to 20.4 mm, with a mean of 6.2 mm. The E-component differences ranged from −23.6 to 12.0 mm, with a mean of −5.1 mm. The U-component differences ranged from −65.3 to 13.3 mm, with a mean of −18.1 mm, while the L-component differences ranged from 3.3 to 27.9 mm, with a mean of 14.1 mm. For Scheme D, the corresponding ranges were −12.4 to 12.0 mm for N, −23.0 to 13.5 mm for E, −48.3 to −2.5 mm for U, and −6.0 to 29.0 mm for L, with mean values of 0.6, −2.0, −18.0, and 14.9 mm, respectively.

Compared with Scheme E, which was used as the relative reference scheme, the four BDS-3 processing schemes (Schemes A–D) showed mean baseline vector component differences at the millimeter level in the north (N) and east (E) components, whereas the mean differences in the up (U) and length (L) components were at the centimeter level. These results indicate that the BDS-3 schemes maintained good horizontal consistency with the GPS L1/L5 reference solution, while the U and L components remained more sensitive to long-baseline error propagation and network-adjustment effects.

(2) Analysis of baseline vector repeatability

**Figure 5:**
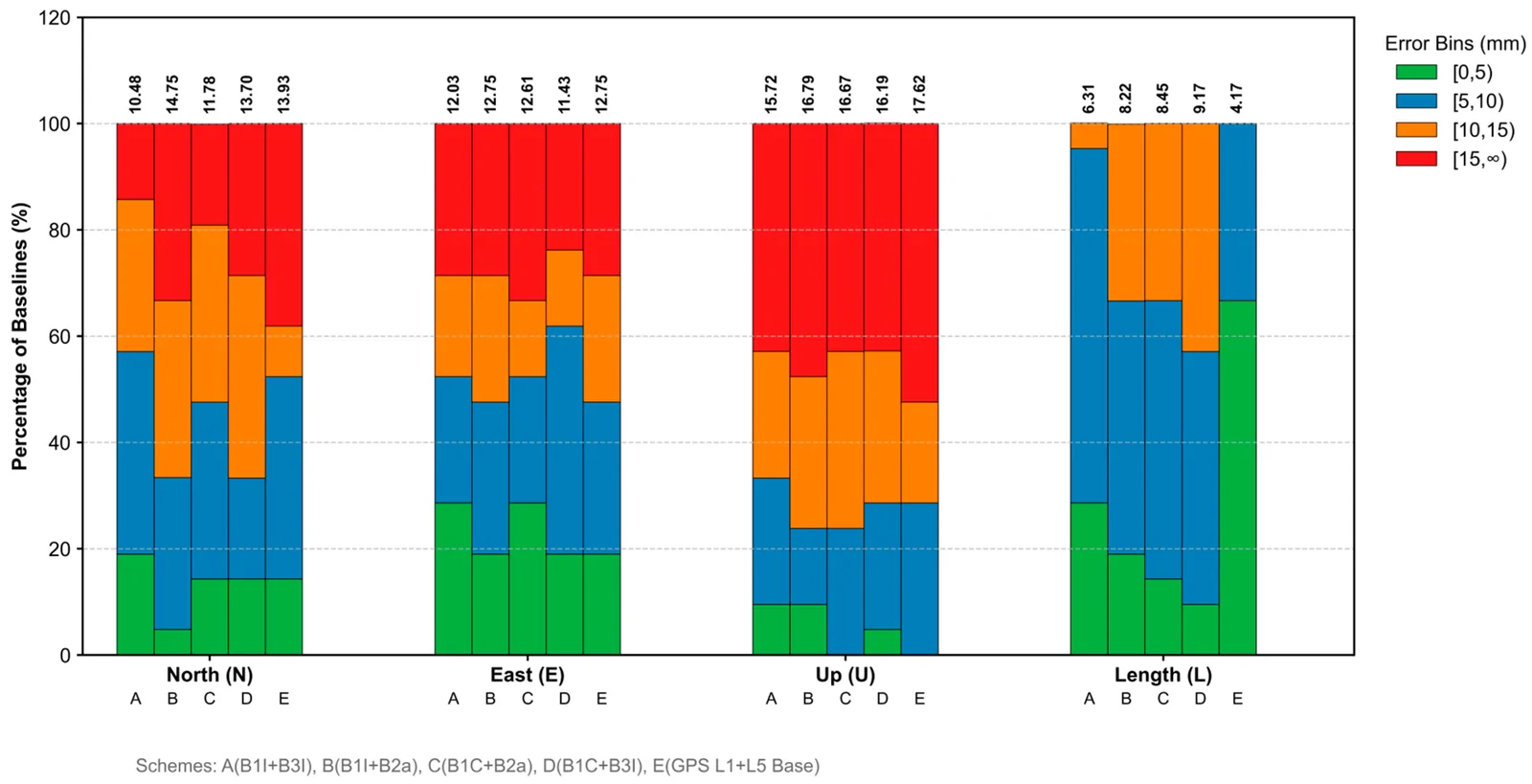
Baseline vector repeatability statistics.

Repeatability analyses were conducted on baseline vectors with the same names from each time period across the five schemes in the north (RN), east (RE), elevation (RU), and length (RL) directions. The statistical results of the baseline vector repeatability analysis are shown in Figure 5.

Specifically, the repeatability characteristics of the five schemes showed differences related to the selected frequency combinations. For the horizontal components (N and E), most baseline repeatability values are concentrated in the millimeter-to-centimeter range, indicating stable horizontal consistency among different frequency combinations. In contrast, the up component (U) shows relatively larger variations for all schemes, reflecting the stronger impact of residual tropospheric errors and atmospheric parameter estimation on the vertical component in long-baseline solutions.

Among the five schemes, Scheme E (GPS L1/L5) achieved the best baseline-length repeatability, with a mean value of 4.17 mm. The corresponding mean length repeatability values for the four BDS-3 schemes, Schemes A–D, were 6.31, 8.22, 8.45, and 9.17 mm, respectively. These results indicate that the modernized GPS L1/L5 combination provides the highest stability for long-baseline distance estimation under the current processing strategy.

Compared with the GPS L1/L5 reference scheme, Scheme E, the four BDS-3 schemes showed broadly comparable repeatability characteristics. The mean repeatability values of the N, E, and L components remain within the millimeter-to-centimeter level, demonstrating that BDS-3 frequency combinations can provide stable baseline vector solutions for long-baseline coordinate transfer. Among these schemes, Scheme A exhibits the smallest length repeatability except for Scheme E, whereas Schemes B–D show similar performance levels, suggesting that the selection of BDS-3 frequency combinations has a limited influence on horizontal baseline stability under the adopted processing conditions.

The relatively larger repeatability values of the U component in the evaluated schemes further indicate that vertical baseline components are more sensitive to unmodeled atmospheric effects. This behavior is consistent with the characteristics of long-baseline GNSS processing, where residual tropospheric delays and parameter correlations mainly affect the vertical component. Overall, the repeatability results demonstrate that the BDS-3 high- and low-frequency combinations achieve stability comparable to the GPS reference schemes, particularly for horizontal baseline components and baseline length determination.

(3) RMS Analysis of Errors in Baseline Vectors

The root mean square (RMS) values of the formal errors of corresponding baseline vectors from different observation sessions were calculated for the entire network. The formal precision of the baseline solutions was evaluated in the north (N), east (E), up (U), and length (L) components. The calculation formula is given as follows:

(23) $$\mathrm{RMS}=\sqrt{\frac{1}{n}\sum_{i=1}^{n}\sigma_{i}^{2}}$$

Table 4 summarizes the RMS values of baseline vector formal errors for the five frequency-combination schemes. The results show clear differences in formal precision among the processing strategies. Scheme C (B1C/B2a), representing the pure new-frequency BDS-3 combination, achieved the lowest formal-error RMS values among the BDS-3 schemes, reaching 2.2, 2.7, 5.8, and 3.1 mm in the N, E, U, and L components, respectively.

Scheme B (B1I/B2a), which also incorporates the B2a signal, exhibited similar performance, with corresponding RMS values of 2.3, 2.7, 6.1, and 3.1 mm. The comparable millimeter-level formal precision of Schemes B and C indicates that the introduction of the B2a signal contributes to improved baseline solution precision under long-baseline conditions.

In contrast, Schemes A (B1I/B3I) and D (B1C/B3I), which use the legacy B3I signal as the low-frequency component, show relatively larger formal-error RMS values, particularly in the E, U, and L components. The GPS L1/L5 reference scheme, Scheme E, showed a larger formal-error RMS in the U component, reaching 14.2 mm, with increased variations also observed in the E and L components.

Overall, the baseline vector formal-error RMS results demonstrate that Scheme C provides the highest internal precision among the evaluated BDS-3 combinations, with reduced random error levels in the experimental network. This improved formal precision provides more reliable baseline vector observations for subsequent three-dimensional constrained network adjustment. However, formal errors mainly describe the internal precision of the baseline solutions and cannot fully represent coordinate-transfer performance. Therefore, a comprehensive assessment should further consider baseline repeatability and externally constrained coordinate-transfer results.

#### 3.2.4. Analysis of Baseline Vector Formal-Error Differences

**Figure 6:**
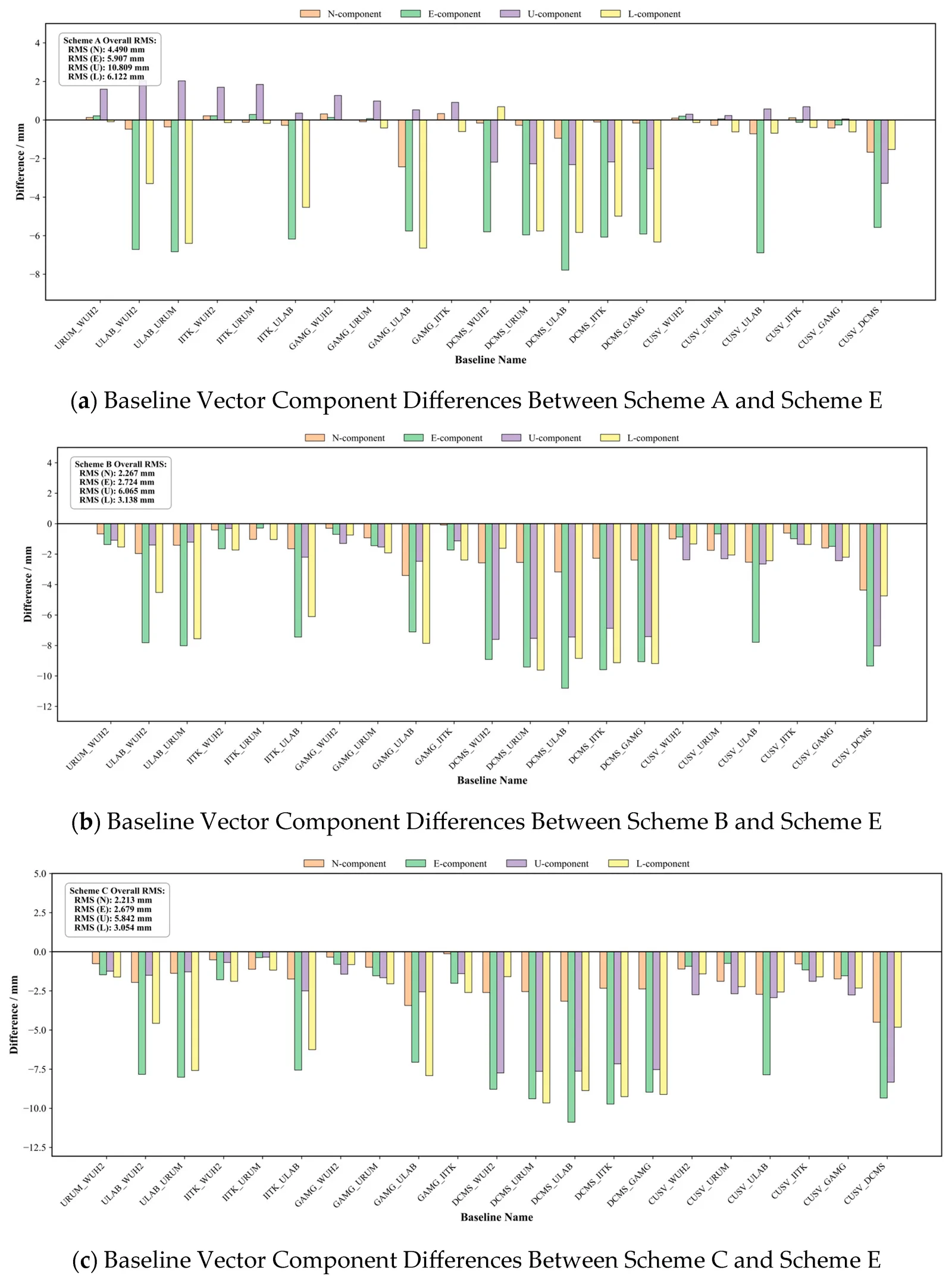

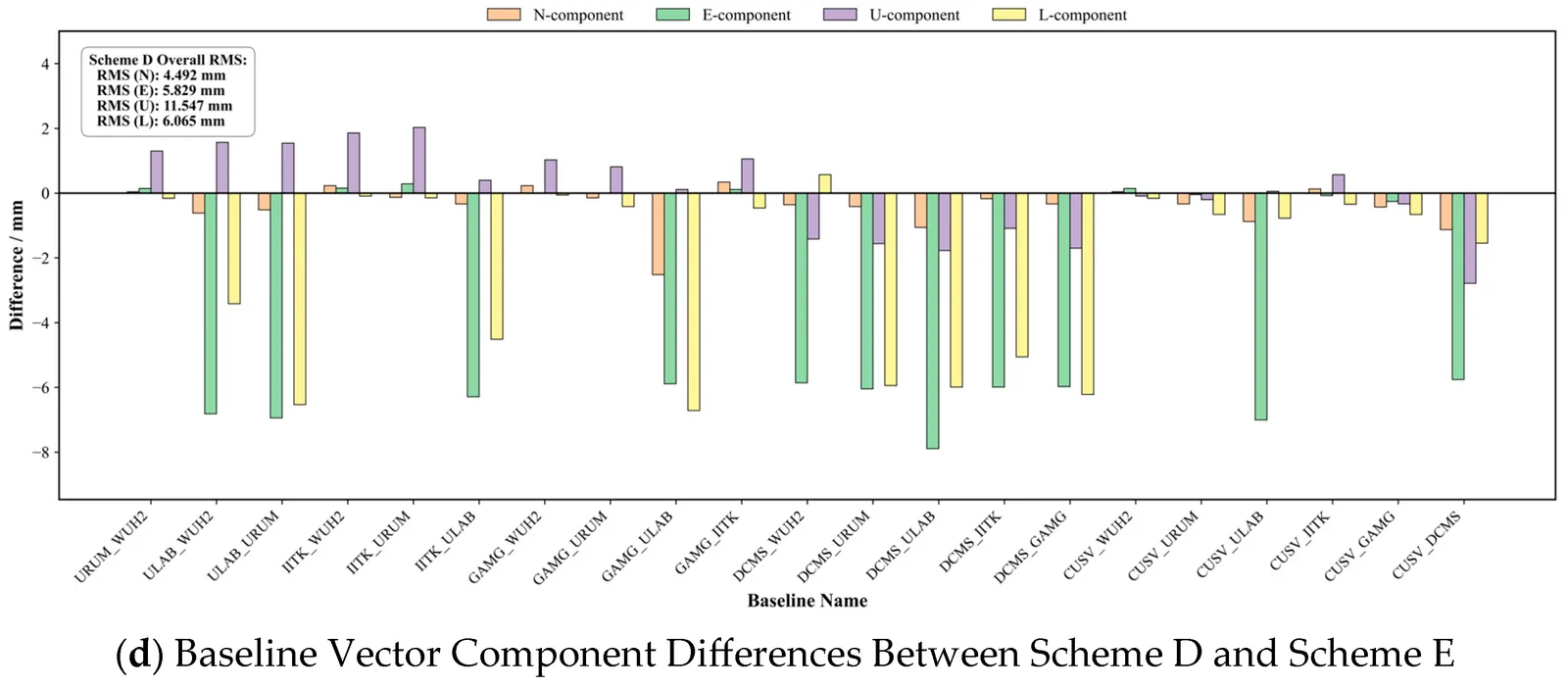
Baseline vector formal-error statistics for the five frequency-combination schemes.

The formal errors of corresponding baseline vectors obtained from the five frequency-combination schemes were averaged over all observation sessions. Using Scheme E (GPS L1/L5) as the relative reference scheme, the differences in baseline vector formal errors were calculated for the north (N), east (E), up (U), and length (L) components. Figure 6 presents the formal-error differences among the five schemes in each component.

Figure 6 presents the baseline vector formal-error differences between Schemes A–D and the GPS L1/L5 reference scheme, Scheme E, in the north (N), east (E), up (U), and length (L) components. The mean formal-error differences were calculated using Scheme E as the relative reference scheme.

As shown in Figure 6, the mean formal-error differences of Scheme A were −0.3, −3.3, 0.0, and −2.3 mm for the N, E, U, and L components, respectively. The corresponding values for Scheme B were −1.7, −5.1, −3.3, and −4.2 mm, respectively, while Scheme C yielded differences of −1.8, −5.1, −3.5, and −4.3 mm, respectively. For Scheme D, the mean differences were −0.4, −3.3, 0.1, and −2.3 mm in the N, E, U, and L components, respectively.

Overall, the formal-error differences of the four BDS-3 schemes relative to Scheme E remained small. The absolute differences were generally within 2.0 mm for the N component and within 4.0 mm for the U component, indicating comparable formal precision between the BDS-3 schemes and the GPS L1/L5 reference solution. In particular, Schemes A and D showed minimal differences in the U component, with absolute values below 0.5 mm.

For the E and L components, Schemes A and D, which do not include the B2a signal, showed relatively smaller formal-error differences, with absolute values within 3.5 mm. In comparison, Schemes B and C, which incorporate the BDS-3 B2a signal, exhibited slightly larger differences in the E and L components, with maximum absolute differences within 5.5 mm and 4.5 mm, respectively. These variations may be associated with differences in signal characteristics, observation noise properties, and frequency-combination performance under long-baseline conditions.

In this study, the baseline vector solutions obtained from the five frequency-combination schemes were systematically compared using component differences, repeatability, and formal-error characteristics. Relative to the GPS L1/L5 reference scheme, the four BDS-3 schemes showed broadly consistent baseline vector behavior. The formal-error indicators further suggest that these schemes can offer reliable observational inputs for subsequent long-baseline position datum coordinate transfer through network adjustment.

## 4. Results

Initially, ULAB was selected as the reference station for the network adjustment. The ITRF2020 coordinates of ULAB at the reference epoch of 5 March 2026 were obtained from the ITRF database and introduced as fixed coordinates in the three-dimensional free network adjustment, while the remaining stations were treated as unknown points. Three-dimensional free network adjustments were then performed using CosaGPS v6.0.

All five frequency-combination schemes successfully produced three-dimensional coordinate estimates for the unknown stations. The corresponding posterior unit weight standard errors were 3.944, 2.716, 2.682, 3.457, and 5.394 cm for Schemes A–E, respectively. These results indicate that all five frequency combinations provided feasible baseline-network solutions with acceptable internal consistency. However, differences in internal adjustment precision were observed among the schemes, reflecting the influence of frequency-combination characteristics on baseline-solution quality.

Subsequently, CUSV, GAMG, IITK, URUM, and ULAB were introduced as fixed control stations for the three-dimensional constrained network adjustment. Their ITRF2020 coordinates at the reference epoch of 5 March 2026 were used as external constraints, while WUH2 was selected as the external check point and DCMS was treated as the unknown station to be determined.

Using Scheme E (GPS L1/L5) as the relative reference scheme and the ITRF2020 coordinates of WUH2 as external validation values, the coordinate-transfer results obtained from the five frequency-combination schemes were compared and are summarized in Table 5.

According to Table 5, the coordinate-transfer performance of the five frequency-combination schemes was evaluated using the known coordinates of the check station WUH2 and the transferred coordinates of the unknown station DCMS.

For WUH2, which was introduced as an external check point, the coordinate differences relative to the known ITRF2020 values were analyzed. The GPS-based schemes (E) exhibited identical three-dimensional differences of −12.6, 30.3, −5.4, and 33.3 mm in the N, E, U, and three-dimensional components, respectively. The BDS-3 schemes showed differences at the centimeter level, with total three-dimensional discrepancies ranging from 48.0 to 64.5 mm. Among the BDS-3 combinations, Scheme C (B1C/B2a) achieved the smallest three-dimensional discrepancy of 48.0 mm, indicating the best consistency with the external reference coordinates.

The coordinate differences relative to Scheme E further reveal the consistency among the BDS-3 frequency combinations and the GPS L1/L5 reference solution. The three-dimensional differences of Schemes A–D relative to Scheme E were 80.7, 64.6, 45.2, and 65.0 mm, respectively. These results indicate that introducing external datum constraints leads to different scale and orientation adjustments among the network solutions. Nevertheless, the BDS-3 frequency combinations maintain stable coordinate-transfer performance under the constrained adjustment framework.

For the unknown station DCMS, the coordinate differences relative to Scheme E were evaluated to assess the compatibility of the four BDS-3 frequency combinations in long-baseline position datum transfer. Schemes A–D showed three-dimensional coordinate differences of 21.6, 28.0, 19.7, and 25.3 mm, respectively, demonstrating centimeter-level consistency with the GPS L1/L5 reference solution. Among them, Scheme C achieved the smallest difference of 19.7 mm, indicating that the B1C/B2a combination provides coordinate-transfer performance comparable to that of the GPS L1/L5 reference scheme.

Overall, the constrained network-adjustment results demonstrate that the BDS-3 frequency combinations, particularly Scheme C (B1C/B2a), can achieve coordinate-transfer performance comparable to that of the GPS L1/L5 reference scheme for high-precision long-baseline position datum transfer.

## 5. Discussion

**Figure 7:**
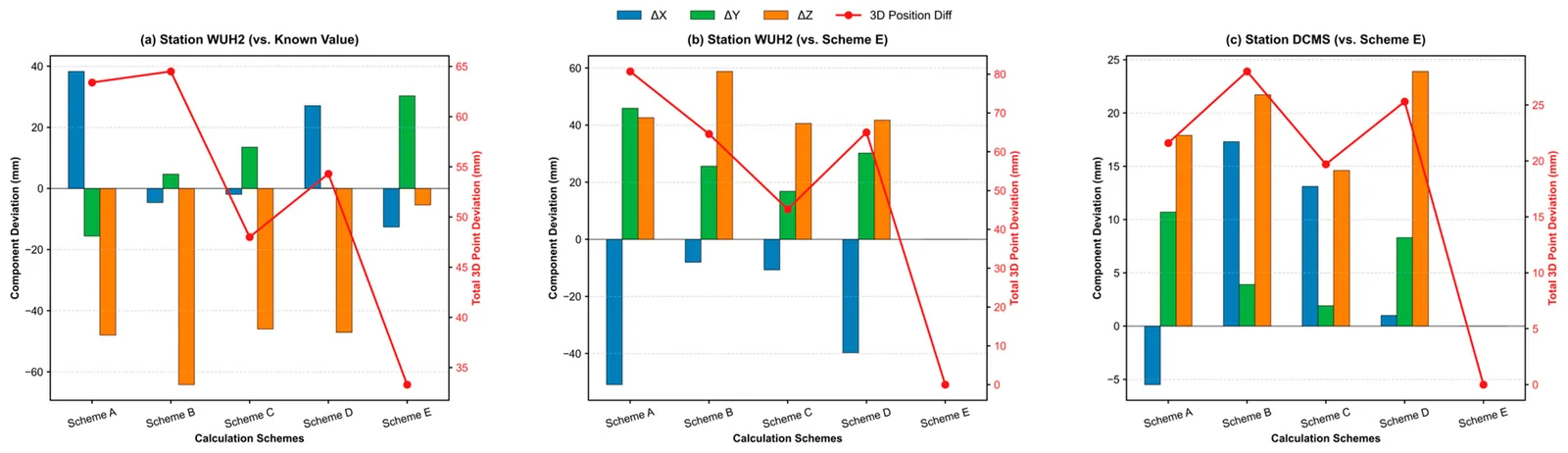
Point differences from the three-dimensional constrained network adjustment.

Based on the network-adjusted point differences listed in Table 5, Figure 7 compares the three-dimensional component differences and total point differences of stations WUH2 and DCMS across the five schemes. WUH2 was evaluated against its known coordinates, whereas DCMS was compared with the GPS L1/L5 reference solution, Scheme E.

As shown in Figure 7, the five schemes produced centimeter-level point differences at the known station WUH2, with absolute three-dimensional differences ranging from 33.3 to 64.5 mm. This result suggests that the five schemes produced broadly comparable point-transfer results at WUH2 under the same network geometry and processing strategy. However, the differences among schemes became more evident at the unknown transfer station DCMS, where Scheme E was used as the reference.

At DCMS, the four BDS-3 schemes showed good consistency with Scheme E. The three-dimensional point differences of Schemes A, B, C, and D were 21.6, 28.0, 19.7, and 25.3 mm, respectively. Among them, Scheme C, based on the BDS-3 B1C/B2a combination, produced the smallest point difference. This result is consistent with its carrier-phase precision and baseline vector formal-error RMS, suggesting that B1C/B2a provides a more stable coordinate-transfer result among the BDS-3 schemes.

This behavior can be partly explained by the lower noise amplification of the B1C/B2a ionosphere-free combination. Scheme C uses B1C and B2a, which are frequency-aligned with GPS L1 and L5, and its ionosphere-free combination noise amplification factor is 2.588. This value is lower than that of the B1I/B3I combination used in Scheme A, whose noise amplification factor is 3.527. A lower noise amplification factor can reduce the propagation of carrier-phase observation noise into the baseline solution, which helps explain the smaller point difference obtained by Scheme C.

By contrast, Schemes A and D do not include the new low-frequency B2a signal. Their ionosphere-free combinations may therefore amplify carrier-phase observation noise more strongly and be more sensitive to unmodeled atmospheric residuals. This can lead to slightly larger differences in the final adjusted coordinates. Overall, the results show that different high- and low-frequency BDS-3 combinations produce a clear performance gradient in long-baseline position datum coordinate transfer, with B1C/B2a showing the best overall consistency among the BDS-3 schemes under the experimental conditions of this study.

## 6. Conclusions and Outlook

This study assessed the applicability of BDS-3 new-frequency signals for high-precision position datum long-baseline coordinate transfer and examined the selection of suitable frequency combinations. An experimental network consisting of seven tracking stations and 21 long baselines was established. Five high- and low-frequency dual-frequency schemes were tested, including four BDS-3 combinations, namely B1I/B3I, B1I/B2a, B1C/B2a, and B1C/B3I, and one GPS reference combination, L1/L5. The evaluation combined double-differenced ionosphere-free baseline processing with three-dimensional constrained network adjustment. Using GPS L1/L5 as the relative reference scheme, the five schemes were compared in terms of carrier-phase precision, normalized root mean square (NRMS), baseline vector quality, and network-adjusted point-transfer results. The main conclusions are as follows.

First, all five schemes achieved millimeter-level carrier-phase precision for the high-frequency signals, with mean values ranging from 6.1 to 7.5 mm. Scheme C, based on the BDS-3 B1C/B2a combination, achieved a mean precision of 6.3 mm, while Scheme B, based on B1I/B2a, achieved 6.4 mm. These values were close to that of the GPS L1/L5 reference scheme, Scheme E, which achieved 6.1 mm, and were better than those of the other BDS-3 combinations, Schemes A and D. These results suggest that the BDS-3 B1C/B2a and B1I/B2a combinations provide stable carrier-phase observation quality under the experimental conditions of this study, with B1C/B2a showing the best carrier-phase precision among the BDS-3 schemes.

Second, the mean NRMS values of all five schemes remained stable at approximately 0.17–0.23, indicating generally reliable baseline solutions for long baselines exceeding 1000 km. Since all schemes were processed as fixed solutions with narrow-lane ambiguity-fixing rates above 90%, these NRMS values characterize the quality of the final biases-fixed solutions. Scheme C had a mean NRMS of approximately 0.23, which was comparable to that of the GPS L1/L5 reference scheme, Scheme E. This result suggests that the BDS-3 B1C/B2a combination is compatible with the long-baseline processing strategy used in this study and can provide a stable observation basis for position datum coordinate transfer.

Third, the four BDS-3 schemes showed good baseline vector consistency relative to the GPS L1/L5 reference scheme. The mean component differences of Schemes A–D were mainly at the millimeter level in the north and east components and at the centimeter level in the up and length components. Baseline vector repeatability also showed that the up component was more affected by residual tropospheric errors over long baselines, with mean repeatability at the centimeter level, whereas the north, east, and length components remained mostly within the millimeter-to-centimeter range. For Scheme C, the baseline vector formal-error RMS values were approximately 2.2, 2.7, 5.8, and 3.1 mm in the north, east, up, and length components, respectively. The east and length components were comparable to those of Scheme B. For baseline vector formal-error differences relative to Scheme E, all schemes remained at the millimeter level, with absolute mean differences within 2.0 mm for the north component, 5.5 mm for the east component, 4.0 mm for the up component, and 4.5 mm for the length component. These results indicate that B1C/B2a provides relatively strong internal consistency and random-error control among the BDS-3 schemes.

Fourth, the three-dimensional constrained network adjustment further supports the feasibility of BDS-3 frequency combinations for position datum transfer. At the known check station WUH2, the absolute point differences of the four BDS-3 schemes relative to the known coordinates ranged from 48.0 to 64.5 mm, with Scheme C giving the smallest difference of 48.0 mm. At the unknown transfer station DCMS, using Scheme E (GPS L1/L5) as the relative reference, the point differences of Schemes A–D were 21.6, 28.0, 19.7, and 25.3 mm, respectively. Scheme C again produced the smallest difference. These results show that the BDS-3 B1C/B2a combination achieved the best overall coordinate-transfer consistency among the BDS-3 schemes under the experimental conditions of this study.

Overall, the BDS-3 B1C/B2a combination showed the best overall consistency among the BDS-3 schemes when carrier-phase precision, NRMS, baseline vector quality, and network-adjusted point-transfer results were considered together. The main contribution of this study is that it extends the evaluation of BDS-3 new-frequency signals from isolated signal-quality analysis to a complete verification chain covering observations, baseline vectors, network adjustment, and coordinate transfer. The results provide preliminary empirical evidence for the use of BDS-3 new-frequency combinations in regional and larger-scale high-precision position datum transfer, subject to validation with longer time series.

This study also has limitations. The dataset covered only seven consecutive days, so it cannot fully represent longer-period effects that affect long-term coordinate-transfer stability, including long-wavelength orbit errors, seasonal variations in tropospheric modeling, nonlinear crustal deformation, geocenter motion, and solar-cycle-related disturbances. In addition, the up component over very long baselines remains affected by residual errors in empirical tropospheric models, which are typically at the 1–2 cm level. Accordingly, the present conclusions should be regarded as preliminary, and the practical applicability of the B1C/B2a combination should be further validated with longer time series. Future work will include cross-validation with alternative processing software (e.g., Bernese GNSS Software), validation of the coordinate-transfer procedure against terrestrial measurements on a smaller network, and a sensitivity analysis of the network configuration and reference-station selection. Future work will also include B2b-based dual-frequency combinations (e.g., B1C/B2b and B1I/B2b), multi-system integrated processing, precise bias corrections, refined stochastic modeling, and longer observation periods to improve the long-term stability and practical applicability of BeiDou-based high-precision position datum transfer. In this regard, the development of dedicated high-precision data processing software platforms capable of accommodating B2b signals, such as further upgrades to GAMIT/GLOBK, will provide a flexible foundation for broadening the current set of frequency combinations.

## Figures and Tables

**Table 1:** Carrier-phase precision statistics for the five frequency-combination schemes.

Monitoring Station	Time Slot	A/mm	B/mm	C/mm	D/mm	E/mm
CUSV	064 d	6.6	6.2	5.5	6.0	6.1
	065 d	10.2	10.0	8.9	7.9	7.3
	066 d	7.5	6.8	6.6	7.7	7.1
	067 d	7.5	10.0	9.6	10.3	8.0
	068 d	9.1	8.2	8.1	9.0	6.9
	069 d	9.8	8.4	9.7	9.6	9.6
	070 d	7.2	6.2	6	7	9.6
	**Mean**	**8.3**	**8.0**	**7.8**	**8.2**	**7.8**
DCMS	064 d	8.2	6.8	7.0	8.7	7.0
	065 d	7.2	6.5	6.5	7.5	6.3
	066 d	7.1	6.1	6.7	7.7	6.3
	067 d	7.1	6.2	6.2	7.5	6.4
	068 d	7.2	5.8	5.7	7.2	6.3
	069 d	7.4	6.2	6.2	7.4	6.4
	070 d	6.4	5.3	5.5	6.7	6.4
	**Mean**	**7.2**	**6.1**	**6.3**	**7.5**	**6.4**
GAMG	064 d	7.0	6.0	5.5	6.4	5.4
	065 d	7.1	5.6	6.0	6.6	4.7
	066 d	6.8	5.8	4.6	5.6	4.4
	067 d	6.8	4.8	4.7	5.9	4.5
	068 d	6.2	5.0	4.7	6.0	4.3
	069 d	6.0	4.8	4.7	5.7	4.3
	070 d	6.0	4.7	4.8	5.9	4.3
	**Mean**	**6.6**	**5.2**	**5.0**	**6.0**	**4.6**
IITK	064 d	6.3	8.2	7.6	7.7	5.4
	065 d	7.9	8.4	7.8	8.6	5.6
	066 d	7.2	8.2	7.9	8.6	5
	067 d	7.2	6.7	6.2	7.5	5.7
	068 d	6.4	6.5	6.2	7.6	5.8
	069 d	7.5	7.5	7.4	8.5	6.7
	070 d	6.2	6.4	5.9	7.2	6.7
	**Mean**	**7.0**	**7.4**	**7.0**	**8.0**	**5.8**
ULAB	064 d	6.7	5.9	5.7	6.4	5.2
	065 d	8.1	6.1	6.5	7.6	5.2
	066 d	7.2	5.1	4.8	6.8	5.1
	067 d	7.2	5.5	5.5	6.7	5.3
	068 d	6.5	5.0	4.9	6.4	5.3
	069 d	6.6	5.0	5.0	6.5	5.4
	070 d	7.1	5.2	4.9	6.7	5.4
	**Mean**	**7.1**	**5.4**	**5.3**	**6.7**	**5.3**
URUM	064 d	8.1	6.4	6.2	8.0	6.5
	065 d	10.1	6.8	7.8	9	6.5
	066 d	8.2	6.7	6.3	7.7	6.5
	067 d	8.2	6.5	6.6	8.1	6.5
	068 d	8.3	6.6	6.6	8.2	6.4
	069 d	8.8	7.0	6.9	8.7	7.0
	070 d	8.6	6.7	6.6	8.3	7.0
	**Mean**	**8.6**	**6.7**	**6.7**	**8.3**	**6.6**
WUH2	064 d	7.8	6.6	7.0	7.6	6.4
	065 d	8.0	7.1	7.2	8.9	6.4
	066 d	7.7	6.0	6.3	7.5	6.0
	067 d	7.7	6.1	6.0	7.5	6.2
	068 d	7.2	5.7	5.8	7.3	5.9
	069 d	7.3	5.9	6.0	7.5	6.3
	070 d	7.0	5.7	5.8	7.0	6.3
	**Mean**	**7.5**	**6.2**	**6.3**	**7.6**	**6.2**
**Mean precision**		**7.5**	**6.4**	**6.3**	**7.5**	**6.1**

**Table 2:** Ambiguity-fixing statistics of the five frequency-combination schemes (DOY 064–070, 2026).

Scheme	Combination	Phase Ambiguities in Solution *	WL Ambiguities Resolved *	WL * Fixing Rate (%)	NL Ambiguities *	NL Ambiguities Fixed *	NL * Fixing Rate (%)
A	B1I/B3I	1121	1008	89.9	951	860	90.4
B	B1I/B2a	1121	821	73.2	773	738	95.5
C	B1C/B2a	1122	839	74.8	784	753	96
D	B1C/B3I	1123	1017	90.6	953	872	91.5
E	GPS L1/L5 (reference)	741	527	71.1	491	462	94.1

* WL: wide-lane; NL: narrow-lane. The statistics are aggregated over the seven daily solutions from DOY 064 to DOY 070 in 2026. “Phase ambiguities in solution” and “WL ambiguities resolved” are obtained from the wide-lane statistics of AUTCLN in the GAMIT q-files; “NL ambiguities” and “NL ambiguities fixed” denote the total and integer-fixed narrow-lane biases, respectively.

**Table 3:** Baseline vector component differences relative to the GPS L1/L5 reference scheme.

Baseline	Scheme	N Component/m	E Component/m	U Component/m	L Component/m	N/mm	E/mm	U/mm	L/mm
CUSV_DCMS	A	2,827,770.0370	1,333,012.4097	−822,470.1016	3,232,593.7782	9.6	8.6	−35.7	21.0
	B	2,827,770.0274	1,333,012.4135	−822,470.1135	3,232,593.7744	0.1	12.3	−47.6	17.3
	C	2,827,770.0302	1,333,012.4132	−822,470.1311	3,232,593.7812	2.9	12.0	−65.3	24.1
	D	2,827,770.0393	1,333,012.4066	−822,470.1141	3,232,593.7821	12.0	5.4	−48.3	25.0
	E	2,827,770.0274	1,333,012.4012	−822,470.0659	3,232,593.7571	0	0	0	0
DCMS_GAMG	A	−392,509.9144	1,047,461.7272	−97,902.9781	1,122,864.6828	−6.0	1.4	−17.5	5.0
	B	−392,509.9181	1,047,461.7230	−97,902.9538	1,122,864.6781	−9.7	−2.7	6.7	0.3
	C	−392,509.9187	1,047,461.7266	−97,902.9472	1,122,864.6811	−10.3	0.9	13.3	3.3
	D	−392,509.9131	1,047,461.7314	−97,902.9750	1,122,864.6860	−4.8	5.6	−14.5	8.2
	E	−392,509.9083	1,047,461.7258	−97,902.9605	1,122,864.6778	0	0	0	0
DCMS_IITK	A	−754,310.2640	−3,360,894.8134	−1,009,352.8266	3,589,344.0974	8.2	−21.3	−33.7	27.6
	B	−754,310.2524	−3,360,894.8079	−1,009,352.8291	3,589,344.0905	19.8	−15.7	−36.1	20.7
	C	−754,310.2518	−3,360,894.8157	−1,009,352.8290	3,589,344.0977	20.4	−23.6	−36.0	27.9
	D	−754,310.2708	−3,360,894.8152	−1,009,352.8206	3,589,344.0988	1.4	−23.0	−27.6	29.0
	E	−754,310.2722	−3,360,894.7921	−1,009,352.7930	3,589,344.0698	0	0	0	0
DCMS_ULAB	A	934,991.0422	−687,873.8960	−105,048.0812	1,165,510.1223	6.1	12.9	2.9	−3.0
	B	934,991.0532	−687,873.9064	−105,048.0942	1,165,510.1384	17.0	2.4.0	−10.1	13.2
	C	934,991.0459	−687,873.9094	−105,048.0887	1,165,510.1339	9.8	−0.6	−4.6	8.6
	D	934,991.0383	−687,873.8953	−105,048.0866	1,165,510.1193	2.2	13.5	−2.5	−6.0
	E	934,991.0362	−687,873.9089	−105,048.0841	1,165,510.1253	0	0	0	0
DCMS_URUM	A	812,780.9064	−2,213,061.3895	−450,255.3753	2,400,204.8701	11.4	−18.6	−8.1	22.5
	B	812,780.9181	−2,213,061.3681	−450,255.3833	2,400,204.8558	23.1	2.8	−16.1	8.3
	C	812,780.9123	−2,213,061.3787	−450,255.3806	2,400,204.8631	17.4	−7.8	−13.4	15.5
	D	812,780.9002	−2,213,061.3914	−450,255.3743	2,400,204.8695	5.2	−20.4	−7.1	21.9
	E	812,780.8950	−2,213,061.3709	−450,255.3672	2,400,204.8476	0	0	0	0
DCMS_WUH2	A	−1,015,710.9650	−184,841.9316	−84,393.1351	1,035,836.6210	−13.5	3.0	8.4	12.0
	B	−1,015,710.9461	−184,841.9443	−84,393.1389	1,035,836.6050	5.4	−9.7	4.6	−4.0
	C	−1,015,710.9543	−184,841.9461	−84,393.1464	1,035,836.6140	−2.8	−11.6	−2.9	5.1
	D	−1,015,710.9639	−184,841.9274	−84,393.1514	1,035,836.6205	−12.4	7.2	−7.9	11.5
	E	−1,015,710.9515	−184,841.9346	−84,393.1435	1,035,836.6090	0	0	0	0
Mean difference/mm	A					2.6	−2.3	−14.0	14.2
	B					9.3	−1.8	−16.5	9.3
	C					6.2	−5.1	−18.1	14.1
	D					0.6	−2.0	−18.0	14.9
	E					0	0	0	0

**Table 4:** Baseline vector formal-error RMS statistics.

Scheme	N RMS/mm	E RMS/mm	U RMS/mm	L RMS/mm
A	4.5	5.9	10.8	6.1
B	2.3	2.7	6.1	3.1
C	2.2	2.7	5.8	3.1
D	4.5	5.8	11.5	6.1
E	5.7	17	14.2	13.6

**Table 5:** Point results from the three-dimensional constrained network adjustment.

Station and Role	Scheme	X/m	Y/m	Z/m	ΔX/mm	ΔY/mm	ΔZ/mm	Point Difference/mm
WUH2 (known point)	A	−2,267,750.3597	5,009,154.4792	3,221,294.3760	38.3	−15.6	−48.0	63.4
	B	−2,267,750.3168	5,009,154.4589	3,221,294.3922	−4.6	4.7	−64.2	64.5
	C	−2,267,750.3195	5,009,154.4501	3,221,294.3740	−1.9	13.5	−46.0	48.0
	D	−2,267,750.3485	5,009,154.4635	3,221,294.3751	27.1	0.1	−47.1	54.3
	E	−2,267,750.3088	5,009,154.4333	3,221,294.3334	−12.6	30.3	−5.4	33.3
	Known value ^#^	−2,267,750.3214	5,009,154.4636	3,221,294.3280	0	0	0	0
	A	−2,267,750.3214	5,009,154.4636	3,221,294.3280	−50.9	45.9	42.6	80.7
	B	−2,267,750.3597	5,009,154.4792	3,221,294.3760	−8.0	25.6	58.8	64.6
	C	−2,267,750.3168	5,009,154.4589	3,221,294.3922	−10.7	16.8	40.6	45.2
	D	−2,267,750.3195	5,009,154.4501	3,221,294.3740	−39.7	30.2	41.7	65.0
	E (reference)	−2,267,750.3120	5,009,154.4721	3,221,294.3316	0	0	0	0
DCMS (unknown point)	A	−2*47.8173	4*7.5604	4*7.1873	−5.5	10.7	17.9	21.6
	B	−2*7.7945	4*7.5536	4*7.1911	17.3	3.9	21.7	28.0
	C	−2*7.7987	4*7.5516	4*7.1840	13.1	1.9	14.6	19.7
	D	−2*7.8108	4*7.5580	4*7.1933	1.0	8.3	23.9	25.3
	E (reference)	−2*7.8118	4*7.5497	4*7.1694	0	0	0	0

“#” represents the known value from https://itrf.ign.fr/en/network/map (accessed on 13 July 2026); “*” represents the omission of five digits.

## Data Availability

The data presented in this study are available in the article. Further inquiries can be directed to the corresponding author.
